# The effects of experience of discrimination and acculturation during pregnancy on the developing offspring brain

**DOI:** 10.1038/s41386-023-01765-3

**Published:** 2023-11-15

**Authors:** Marisa N. Spann, Kiarra Alleyne, Cristin M. Holland, Antonette Davids, Arline Pierre-Louis, Claire Bang, Victoria Oyeneye, Rebecca Kiflom, Eileen Shea, Bin Cheng, Bradley S. Peterson, Catherine Monk, Dustin Scheinost

**Affiliations:** 1 Vagelos College of Physicians and Surgeons, Columbia University, New York, NY USA; 2 New York State Psychiatric Institute, New York, NY USA; 3 Columbia University Mailman School of Public Health, New York, NY USA; 4 Rutgers University, Newark, NJ USA; 5 Columbia University, New York, NY USA; 6 Institute for the Developing Mind, Children’s Hospital Los Angeles, Los Angeles, CA USA; 7 Department of Psychiatry, Keck School of Medicine, University of Southern California, Los Angeles, CA USA; 8 Yale University School of Medicine, New Haven, CT USA

**Keywords:** Cognitive neuroscience, Risk factors

## Abstract

The experience of ethnic, racial, and structural inequalities is increasingly recognized as detrimental to health, and early studies suggest that its experience in pregnant mothers may affect the developing fetus. We characterized discrimination and acculturation experiences in a predominantly Hispanic sample of pregnant adolescent women and assessed their association with functional connectivity in their neonate’s brain. We collected self-report measures of acculturation, discrimination, maternal distress (i.e., perceived stress, childhood trauma, and depressive symptoms), and socioeconomic status in 165 women. Then, we performed a data-driven clustering of acculturation, discrimination, perceived stress, depressive symptoms, trauma, and socioeconomic status variables during pregnancy to determine whether discrimination or acculturation clustered into distinct factors. Discrimination and acculturation styles loaded onto different factors from perceived stress, depressive symptoms, trauma, and socioeconomic status, suggesting that they were distinct from other factors in our sample. We associated these data-driven maternal phenotypes (discrimination and acculturation styles) with measures of resting-state functional MRI connectivity of the infant amygdala (*n* = 38). Higher maternal report of assimilation was associated with weaker connectivity between their neonate’s amygdala and bilateral fusiform gyrus. Maternal experience of discrimination was associated with weaker connectivity between the amygdala and prefrontal cortex and stronger connectivity between the amygdala and fusiform of their neonate. Cautiously, the results may suggest a similarity to self-contained studies with adults, noting that the experience of discrimination and acculturation may influence amygdala circuitry across generations. Further prospective studies are essential that consider a more diverse population of minoritized individuals and with a comprehensive assessment of ethnic, racial, and structural factors.

## Introduction

The history of the United States is marked by ethnic, racial, and structural inequalities [1–3]. Beyond this, the United States’ history with immigration, and thus acculturation within immigrant populations, is nearly as old as the country itself [4]. Despite the rapidly growing foreign-born population in the United States [5], stressors related to ethnic, racial, and structural inequalities continue to contribute to adverse physical and mental health effects of immigrants and populations of color [6, 7]. The everyday experience of discrimination—the unjust treatment of individuals based on characteristics such as ethnic or racial group, gender, age, or sexual orientation [8]—and acculturative stress—the demands, strains, and ‘wear and tear’ of acculturation, or the process of adapting to a different culture [9]—are potential stressful experiences that can lead to adverse effects and the primary variables of interest for this study. Our understanding of how individuals may perceive these experiences is complex. It has been suggested that these experiences have varied effects on the health of individuals in prior studies [10].

The effects of discrimination and acculturation during pregnancy can have profound ramifications on offspring. Experiences of discrimination can be detrimental to the psychological well-being of pregnant women [11], and they are associated with increased infant mortality [12, 13], preterm birth [14], and decreased infant birth weight [15]. Similar experiences of acculturation are associated with depression [16–20], anxiety [21, 22], and stress, particularly acculturative stress [23, 24], in pregnant women. These experiences have unique and specific effects on psychological well-being that are not solely moderated or mediated by stress or depression [25]. Despite the profound impact these experiences can have on pregnant woman of color and their fetuses, scant research has characterized the impact of these experiences on brain-based outcomes in offspring. However, the comparative literature on outcomes in adults is more robust [10, 26–35].

Studies are beginning to demonstrate the neural correlates of discrimination, acculturation, and ethno-racial processing in adults [36–38]. Experiences of discrimination correlate with stronger resting state functional connectivity between the amygdala and several brain regions, including the frontal lobe [39]. The amygdala appears involved in automatic evaluation of discrimination and acculturation experiences [40]. Maternal discrimination and acculturation stressors have not explicitly been explored in relation to brain connectivity in infant offspring. However, studies of other prenatal stressors suggest that amygdala connectivity is a good candidate for investigating brain outcomes associated with these exposures [41–44]. Similar stressors can have unique biological pathways affecting the fetus [45, 46]. For example, perceived stress, anxiety, and depression can uniquely impact the newborn brain [47]. Thus, discrimination and acculturation stressors may have differential impacts on offspring outcomes than other stressors, a consideration the current study evaluates.

About half of the foreign-born population in the United States is of Latin American origin [48], making it a key population of interest to researchers working toward a better understanding of the effects of acculturation [49, 50]. Acculturative stress can negatively affect first, second, and third-generation immigrants [51]. The present study sought to better characterize discrimination and acculturation styles during pregnancy and their associated downstream effects on infant offspring. Our sample of convenience was predominantly Hispanic (88%) adolescent participants. To assess maternal experiences during pregnancy, we collected two measures of acculturation, an experience of discrimination scale, additional measures of maternal distress (i.e., perceived stress, childhood trauma, and depressive symptoms), and an index of socioeconomic status (i.e., income). First, we performed a data-driven clustering of acculturation, discrimination, perceived stress, depressive symptoms, trauma, and socioeconomic status during pregnancy to derive phenotypes distinct from other stressors. Next, we associated these data-driven phenotypes with measures of resting-state functional connectivity of the amygdala in the infants (*n* = 38) within 6 weeks following birth. Finally, we performed exploratory analyses associating the discrimination and acculturation stressors with measures of fetal development (i.e., head circumference; *n* = 92) and birth outcome data (i.e., gestational age at birth and Apgar score at 5 min; *n* = 155). We hypothesized that maternal experience of discrimination and acculturation would be associated with offspring individual differences in amygdala functional connectivity. The novelty of this research precluded specific hypotheses about the direction of these effects.

## Methods and materials

### Participants

One hundred sixty-five nulliparous pregnant women, aged 14 to 19 years, were recruited through the Department of Obstetrics and Gynecology at Columbia University Irving Medical Center (CUIMC), Weill Cornell Medical College, and flyers posted in the CUIMC vicinity as a part of a prospective study from 2009 to 2012 examining the adolescent pregnancy behaviors and infant outcomes. The pregnant adolescents received routine prenatal care and had no major health problems at the time of recruitment. Participating women were excluded if they acknowledged the use of recreational drugs, tobacco, alcohol, or medications that affect cardiovascular functions or lacked fluency in English. The women provided informed written consent for themselves and their infants to participate in the study. For a subset, infants were imaged within the first 6 weeks of life (*n* = 38). Prenatal electronic health records were reviewed to collect ultrasound data and determine birth outcomes (*n*’s = 92–155). The New York State Psychiatric Institute Institutional Review Board approved all study procedures. The sample was predominantly Hispanic/Latinx.

### Self-reported measures

We collected several measures of acculturation, discrimination, and distress during pregnancy. Detailed information about each measure can be found in the Supplementary.

For acculturation, we collected the Acculturation, Habits, and Interests Multicultural Scale for Adolescents (AHIMSA) and the Short Acculturation Scale for Hispanics (SASH) during the 2nd or 3rd trimester (24–37 weeks of gestation). The AHIMSA measures integration (the identification with both cultures), assimilation (identification with the host culture), separation (identification with home culture), and marginalization (the identification with neither home nor host culture). The SASH measures high versus low assimilation. Self-reported language use—a standard proxy for acculturation—was also collected.

For discrimination, we collected the Experiences of Discrimination (EOD). The Experience of Discrimination (EOD) instrument measures self-reported experiences of discrimination and was scored by counting the number of situations for which a participant experienced discrimination [52]. EOD was collected at three time points during pregnancy: at 12–14, 24–26, and 34–36 weeks of gestation.

For distress, we collected the Perceived Stress Scale (PSS), the Reynold’s Adolescent Depression Scale (RADS), and the Childhood Trauma Questionnaire for distress. The PSS, and RADS were collected at three time points during pregnancy: at 12–14, 24–26, and 34–36 weeks of gestation. The CTQ was collected in the third trimester.

Self-reported family/self-income was collected as a 6-point ordinal variable (1: $0–$15,000, 2: $16,000–$25,000, …) as a measure of socioeconomic status.

### Fetal and birth outcomes

We attained fetal morphometric measures and birth outcomes from participants’ electronic health records. Fetal morphometric measures were head circumference (HC) and biparietal diameter (BDP) collected between 16- and 40-weeks gestation. HC was measured along the outer perimeter of the calvaria, parallel to the biparietal diameter. BDP was measured from the outer margin of the proximal skull table to the inner margin of the distal skull table. Participants with two or more ultrasonography records were included to construct growth curves of the two primary fetal outcomes [53]. Birth outcomes were gestational age at birth and Apgar score at 5 min.

### Seed connectivity

After preprocessing (as described in [54, 55] and the Supplementary), we assessed whole-brain seed to voxel connectivity from the right and left amygdala combined into a single seed (Fig. S1) using BioImage Suite [56]. The right and left seeds were manually defined on the reference brain. The time course of the reference region in each participant was then computed as the average time course across all voxels in the seed region. This time course was correlated with the time course for every other voxel in gray matter to create a map of *r*-values, reflecting seed-to-whole-brain connectivity. These *r*-values were transformed to *z*-values using Fisher’s transform, yielding one map for each seed, representing the strength of correlation with the seed for each participant.

### Factor analysis

We performed an exploratory factor analysis to investigate the associations between discrimination, acculturation, and distress during pregnancy. The purpose of our factor analyses was twofold: (1) to identify whether the combination of other stressors was similar to or distinct from the discrimination and acculturation and (2) reduce the dimensions of the discrimination and acculturation collected. Using the R psych package, Eigenvalues were first examined to determine the appropriate number of factors; then, principal component analysis was conducted using Spearman’s or Pearson’s correlations. No rotations were used, and ordinary least squares was used to extract the factors. The integration and separation subscales of the AHIMSA were reverse coded to ensure only positive factor loadings. The scores of scales collected at multiple timepoints (i.e., PSS) were highly correlated (*r* > 0.5) and, thus, were averaged across trimesters into a single score used in analyses. The sum of all CTQ subscales was used. Self-reported family/self-income was included as a measure of socioeconomic status. Factor scores were calculated from the final model and used in subsequent primary analyses. For face validity, we investigated if language use—a standard proxy for acculturation—correlates with the acculturation factors (see Supplementary).

### Statistical analyses

To assess the associations of the variable of interest with potential confounding variables, demographic and behavioral data were analyzed using standard *χ*^2^ test statistics or Fisher exact test for categorical data. Continuous data were analyzed using *t*-tests or Mann–Whitney *U* tests when a normal distribution could not be assumed to compare groups. Linear regression was used to associate fetal growth and birth outcomes with the discrimination and acculturation stressors. The dependent variables were performed with the three latent factors of discrimination and acculturation stressors (derived from the factor analysis) and discrimination as independent variables. The three latent factors of discrimination and acculturation stressors (derived from the factor analysis) were also associated to language use at home. Both unadjusted analyses and analyses adjusted by relevant covariates were performed. *p* values were two-sided. Bonferroni correction was used for non-imaging results. All analyses were performed using SPSS statistical software version 25 (IBM) or SAS statistical software version 9.3 (SAS Institute).

Imaging data were analyzed using voxel-wise linear models controlling for sex and postmenstrual age at scan included in a single model. For the primary analysis, the dependent measure was whole-brain functional connectivity of the amygdala. The independent variables were the acculturation factors and EOD. For analysis with EOD, EOD and imaging data were converted to ranks to account for skewness. Secondary analyses were performed to control for maternal depression and perceived stress. Imaging results were considered significant at *p* < 0.05, corrected for multiple statistical comparisons across gray matter using cluster-level correction [57, 58]. Cluster sizes were determined using AFNI’s 3dClustSim (version 16.3.05) with 10,000 iterations, an initial cluster forming threshold of *p* < 0.001, the gray matter mask applied in preprocessing, and a mixed-model spatial autocorrelation function (ACF). Parameters for the spatial ACF were estimated from the residuals of the voxel-wise linear models using 3dFWHMx. The latent factors of discrimination and acculturation stressors (derived from the factor analysis) were used as independent variables while controlling for the neonate’s postmenstrual age at scan and sex.

## Results

### Demographic characteristics

The maternal and neonatal demographic characteristics for the entire sample, ultrasound sample, and neonatal MRI sample are summarized in Table 1. Demographic characteristics for the total sample did not differ significantly from the subsample with MRI data. All women were adolescents with an average age of 18 years at the time of delivery. Most pregnant women were identified as Hispanic or Latinx (88.48%). Discrimination and acculturation stressors were similar between Hispanic and non-Hispanic individuals (see Supplementary). Ethnic and racial data are summarized in Table 2. Self-report measures of acculturation, discrimination, and other stressors are summarized in Table 3. Importantly, based on self-report data, all women in this study endorsed being a woman of color or underrepresented individual.

**Table 1:** Maternal and neonatal demographics of sample with discrimination and acculturation data.

Variables	Factor analysis and birth outcome sample (*n* = 165)		Sample with MRI data (*n* = 38)		Comparison between samples
	*N*	Mean (SD) or %	*N*	Mean (SD) or %	*T* or $${\chi }^{2}$$ statistic (*p* value)
Maternal					
Age at delivery, years	151	18.25 (1.35)	38	18.11 (1.47)	0.53 (0.60)
Pre-pregnancy body mass index (BMI)	163	25.84 (6.96)	37	24.70 (6.47)	0.92 (0.36)
Years of education	163		37		
8th grade	5	3.07%	0	0.00%	0.26 (0.61)
9th grade	17	10.43%	5	13.51%	0.26 (0.61)
10th grade	20	12.27%	7	18.92%	0.06 (0.80)
11th grade	45	27.61%	7	18.92%	0.84 (0.36)
12th grade or higher	76	46.63%	18	48.65%	0.02 (0.88)
Family income	153		34		0.98 (0.32)
$0–$15,000	68	44.44%	15	44.11%	0.12 (0.72)
$16,000–$25,000	49	32.03%	13	38.23%	0.42 (0.51)
$26,000–$50,000	27	17.64%	4	11.76	5.53 (0.02)
$51,000–$100,000	6	3.92%	6	17.64%	0.47 (0.50)
$101,000–$250,000	2	1.12%	0	0.00%	0.23 (0.63)
>$250,000	1	0.61%	0	0.00%	
Type of delivery	141		34		0.65 (0.42)
Vaginal spontaneous	49	34.75%	15	44.12%	0.30 (0.58)
Assisted vaginal	60	42.55%	12	35.29%	0.01 (0.92)
Emergent C-section	32	22.70%	7	20.59%	
Pregnancy complications	141		32		2.61 (0.11)
None	102	72.34%	29	90.63%	3.59 (0.06)
Complications	39	27.66%	3	9.38%	0.31 (0.76)
Neonatal					
Gestational age at birth, weeks	155	39.11 (2.11)	38	39.22 (1.28)	0.44 (0.66)
Birth weight, gms	150	3158.09 (542.87)	37	3116.42 (442.56)	0.18 (0.86)
Birth head circumference, cms	116	33.93 (1.22)	25	33.89 (1.47)	1.50 (0.14)
Apgar 1 min	141	8.37 (1.36)	34	8.71 (0.68)	0.84 (0.40)
Apgar 5 min	141	8.84 (0.72)	34	8.94 (0.24)	
Postmenstrual age at scan	–	–	38	42.31 (1.56)	0.98 (0.32)
Gender	157		38		
Female	65	41.40%	13	34.21%	0.68 (0.17)
Male	92	58.60%	25	65.79%	0.39 (0.53)

All mothers of Hispanic ethnicity were coded together.

*cms* centimeters, *gms* grams.

**Table 2:** Ethnic and racial data.

Variables	Factor analysis and birth outcome sample (*n* = 165)		Sample with MRI data (*n* = 38)		Comparison between samples
	*N*	%	*N*	%	${\chi }^{2}$ statistic (*p* value)
Ethnicity					
Not Hispanic/Latina	19	11.52%	5	13.16%	0.00 (0.99)
Hispanic/Latina	146	88.48%	33	86.84%	0.02 (0.96)
Race (Hispanic/Latina group)					
American Indian/Native Alaska	85	51.5%	15	39.5%	1.34 (0.25)
Asian	2	1.2%	0	0.0%	0.47 (0.50)
African American/Black	7	4.2%	0	0.0%	0.64 (0.42)
Native Hawaiian/Pacific Islander	2	1.2%	0	0.0%	0.47 (0.50)
White/White	12	7.3%	1	2.6%	0.47 (0.49)
Other	38	23.0%	17	44.7%	6.31 (0.01)
Race (Not Hispanic/Latina group)					
American Indian/Native Alaska	1	0.6%	0	0.0%	0.23 (0.63)
Asian	0	0.0%	0	0.0%	0.00 (1.00)
African American/Black	16	9.7%	5	13.2%	0.11 (0.74)
Native Hawaiian/Pacific Islander	0	0.0%	0	0.0%	0.00 (1.00)
White/White	0	0.0%	0	0.0%	0.00 (1.00)
Other	2	1.2%	0	0.0%	0.47 (0.50)

**Table 3:** Measures of acculturation, discrimination, and distress.

Variables	Factor analysis and birth outcome sample (*n* = 165)		Sample with MRI data (*n* = 38)		Comparison between samples
	*N*	Mean (SD) or %	*N*	Mean (SD) or %	*T* statistic (*p* value)
Acculturation, Habits, and Interests Multicultural Scale for Adolescents (AHIMSA)					
Assimilation	157	2.22 (2.31)	38	2.79 (2.04)	1.40 (0.16)
Separation	157	1.01 (1.41)	38	0.76 (1.02)	1.03 (0.30)
Integration	157	4.55 (2.23)	38	4.29 (1.71)	0.67 (0.50)
Marginalization	157	0.2 (0.65)	38	0.13 (0.41)	0.63 (0.53)
Short Acculturation Scale for Hispanics (SASH)					
SASH	163	3.26 (0.77)	38	3.34 (0.65)	0.59 (0.55)
Experiences of Discrimination (EOD)					
EOD	165	1.69 (2.86)	38	0.97 (1.91)	1.48 (0.14)
Perceived Stress Scale (PSS)					
PSS	165	26.95 (6.04)	38	27.27 (6.06)	0.29 (0.77)
Childhood Trauma Questionnaire (CTQ)					
CTQ	165	35.90 (15.50)	38	39.76 (18.43)	1.33 (0.18)
Reynolds Adolescent Depression Scale (RADS)					
RADS	165	40.39 (13.60)	38	41.23 (13.35)	0.34 (0.73)

*SASH* Short Acculturation Scale for Hispanics, *EOD* experience of discrimination, *PSS* Perceived Stress Scale, *CTQ* Childhood Trauma Questionnaire, *RADS* Reynold’s Adolescent Depression Scale.

### Factor analysis of discrimination and acculturation stressors

Our initial factor analysis included the 4 subscales of the AHIMSA (integration, assimilation, separation, and marginalization), the SASH, the EOD, the PSS, the CTQ, family/self-income, and the RADS. A correlation matrix of all these variables is shown in Fig. S2. Based on the elbow of the Scree plot of the eigenvalues, a 4-factor model was optimal (Fig. S3A). From this analysis, the CTQ, PSS and RADS scores clustered into a single factor; scores from the AHIMSA and the SASH clustered into three acculturation factors; and EOD and income did not cluster into a factor (Fig. S3B). The items and loadings are shown in Table S1. These results suggest that distress and discrimination are single constructs distinct from acculturation for the sample at hand and that acculturation clusters in multiple factors.

**Fig. 1:**
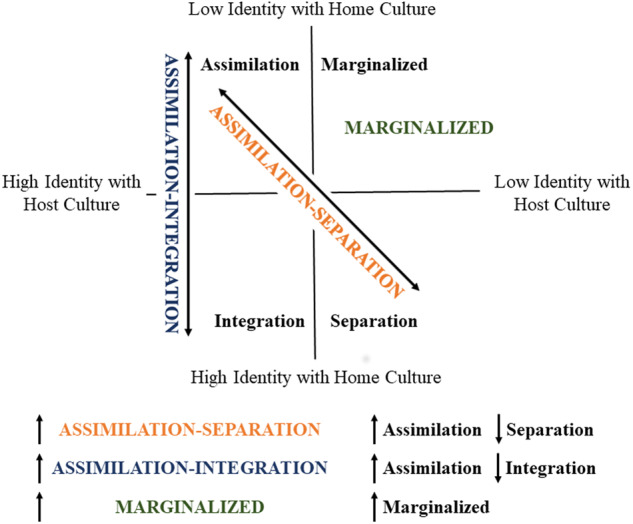
Acculturation factors projected onto Berry’s model of acculturation. In Berry’s multidimensional model of acculturation (see Supplementary), not all four types may be present in any given population [93], which is reflected in our factor analyses. ASSIMILATION-INTEGRATION and ASSIMILATION-SEPARATION each project into two categories with Berry’s model of acculturation. Higher scores in either factor converge to higher assimilation, but lower scores on ASSIMILATION-INTEGRATION and ASSIMILATION-SEPARATION diverge to separation and integration, respectively. MARGINALIZED reflected only the marginalized type of acculturation.

Given this, we performed a factor analysis including only the AHIMSA and the SASH. A 3-factor model was optimal and produced a nearly identical factor structure to the previous model (Fig. S3C, D and Table S2). From the AHIMSA, low integration and high assimilation clustered into a factor (labeled ASSIMILATION-INTEGRATION factor). High levels of ASSIMILATION-INTEGRATION factor reflect a pregnant woman who has come to identify with the host culture (assimilation) rather than integrating both cultures (integration). Separation from the AHIMSA and the SASH clustered into a factor (labeled ASSIMILATION-SEPARATION factor). Higher scores on the ASSIMILATION-SEPARATION factor reflect a pregnant woman who has come to identify with the host culture (assimilation) rather than their home culture (separation). Finally, marginalization loaded onto its own factor (labeled MARGINALIZED factor). Of note, both higher scores on the ASSIMILATION-INTEGRATION factor and higher ASSIMILATION-SEPARATION factor indicate a higher level of assimilation (i.e., identifying with the host culture). While these factors as similar in the positive direction, they differ in the negative direction. Lower scores on the ASSIMILATION-INTEGRATION factor indicate integration of home and host cultures, whereas lower scores on the ASSIMILATION-SEPARATION factor indicate higher identification with home culture. Figure 1 places the three derived factors in the context of the four acculturation categories (i.e., assimilation, separation, integration, and marginalization). Together, these results suggest that the discrimination and acculturation stressors represent distinct factors from other stressors. Further investigations of these factors are presented in the Supplementary.

### Maternal experiences of acculturation associates with amygdala connectivity

**Fig. 2:**
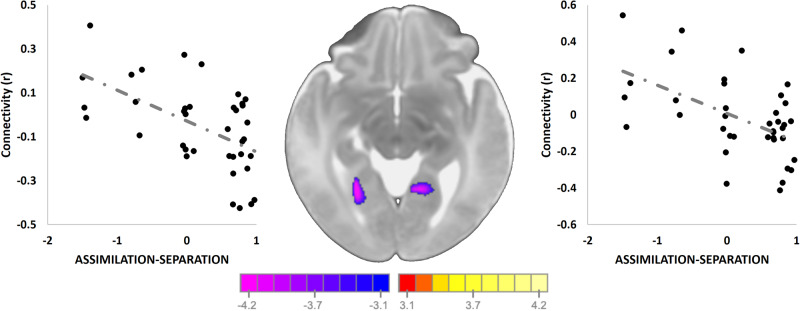
Associations between prenatal acculturation factors and offspring amygdala connectivity. Higher ASSIMILATION-SEPARATION in mothers during pregnancy was associated with weaker infant connectivity between the amygdala and bilateral fusiform gyrus in in their offspring. No significant correlations between the ASSIMILATION-INTEGRATION factor and amygdala connectivity were observed. Scatterplots next to the images visualize the distribution of the observed data points for average infant connectivity in the detected regions plotted against the ASSIMILATION-SEPARATION factor. Models adjusted for other stressors (PSS, RADS, CTQ, SES) remained significant.

We associated the discrimination and acculturation stressors with offspring amygdala connectivity during the neonatal period. As only four women in the imaging sample endorsed experience of marginalization, the MARGINALIZED factor was dropped from this analysis. Higher ASSIMILATION-SEPARATION factor in mothers was associated with weaker connectivity between the amygdala and bilateral fusiform gyrus (*p* < 0.05; Fig. 2 and Table S3) in their offspring. No significant correlations between the ASSIMILATION-INTEGRATION factor and amygdala connectivity were observed. Models adjusted for other stressors (PSS, RADS, CTQ, SES) remained significant.

### Maternal experiences of discrimination associates with amygdala connectivity

**Fig. 3:**
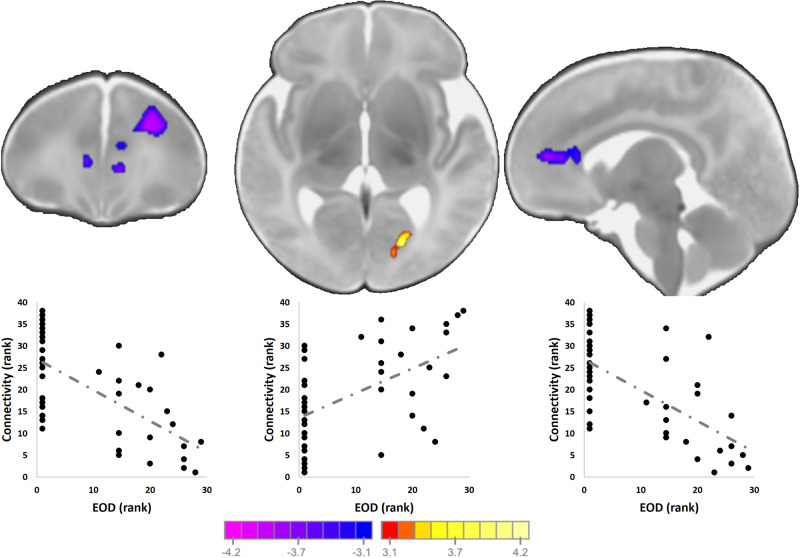
Associations prenatal experiences of discrimination and offspring amygdala connectivity. Neonates born to mothers experiencing discrimination had weaker connectivity between the amygdala and the medial and anterior prefrontal cortices and stronger connectivity the amygdala and the left fusiform gyrus. Scatterplots next to the images visualize the distribution of the observed data points for average infant connectivity in the detected regions plotted against EOD. Models adjusted for other stressors (PSS, RADS, CTQ, SES) remained significant.

Neonates born to mothers experiencing discrimination had weaker connectivity between the amygdala and the medial and anterior prefrontal cortices (*p* < 0.05 corrected; Fig. 3 and Table S3) and stronger connectivity the amygdala and the left fusiform gyrus. Given the skewness of the EOD, we also dichotomized it to those experiencing discrimination and those not and repeated linear modeling. Results were similar. Models adjusted for other stressors (PSS, RADS, CTQ, SES) remained significant.

### Exploratory: discrimination and acculturation stressors and fetal/birth outcomes

We investigated associations of discrimination and acculturation stressors on fetal growth (HC and BPD) and birth outcomes (gestational age at birth and Apgar score at 5 min). After correcting for multiple comparisons, none of the discrimination and acculturation stressors associated with these measures (Tables S4 and S5). The associations between the ASSIMILATION-INTEGRATION factor and slower BPD growth (Cohen’s *D* = 0.43), the ASSIMILATION-INTEGRATION factor and lower Apgar score at 5 min (Cohen’s *D* = 0.33), and the ASSIMILATION-SEPARATION factor and lower gestational age at birth (Cohen’s *D* = 0.34) were the only associations showing an effect size >0.30. Models adjusting for maternal demographic variables (maternal age and c-section, ethnicity, and pre-pregnancy body mass index) and other stressors (PSS, RADS, CTQ, SES) produce similar results.

## Discussion

We investigated the structure of discrimination and acculturation-related factors during pregnancy and their effects on fetal and infant development in a predominantly Hispanic sample of adolescent mothers. Consistent with our hypothesis, discrimination and acculturation loaded onto different factors and independent of stressors (perceived stress, depressive symptoms, trauma, and socioeconomic status), suggesting that discrimination and acculturation factors are distinct from other factors considered stressors in our sample. Higher assimilation was associated with weaker amygdala and bilateral fusiform gyrus connectivity. Maternal experience of discrimination was associated with weaker connectivity between the amygdala and prefrontal cortex and stronger connectivity between the amygdala and fusiform gyrus. In exploratory analyses, the latent factors of discrimination and acculturation did not significantly associate with fetal growth, Apgar scores at 5 min, or gestational age at birth. Together, these results suggest that discrimination and acculturation factors are likely distinct from other stress-related factors, and they may alter fetal brain development.

Functional neuroimaging research has identified the amygdala as being involved in processing in-group and out-group membership, as well as ethnic or racial groups [40, 59, 60]. Our finding that maternal experiences of both acculturation and discrimination associated with amygdala-fusiform connectivity in infant offspring is intriguing. While it is difficult to compare our results directly to the adult literature, previous literature demonstrates an association between the fusiform and ethnic or racial processing in adults [61–65]. Amygdala-fusiform connectivity also plays a role in ethnic or racial processing of faces [60, 62, 66]. At 3 months of age, infants can begin discriminating between the faces of their own and other discrimination and acculturation groups as the “other-race effect” develops [67]. As in adults [63], familiarity causes infants to prefer faces of their discrimination and acculturation group to faces of others [68], but greater exposure to other discrimination and acculturation groups mitigates this preference [69]. Further, studies on young infants have shown that postnatal environmental exposures to people of different discrimination and acculturation groups and cultures affect infants’ perception [70].

Our findings may indicate that infants with mothers who report increased identification with their home culture are mainly exposed to faces of their discrimination and acculturation groups during early postnatal life. In contrast, greater maternal assimilation into the host culture may provide the infant greater exposure to other discrimination and acculturation faces. This early life exposure to other discrimination and acculturation groups’ faces may induce changes to brain function related to discrimination and acculturation processing (such as differences in amygdala-fusiform connectivity) before external behaviors are observable. Nevertheless, follow-up data during infancy would be needed to test these potential links and interweave other types of stimuli as control conditions, such as objects or non-human faces.

Broadly, a common sequela of early life adversity originating in the pre- and post-natal environment is altered amygdala-frontal circuitry [71–77]. Our results are consistent with previous reports of prenatal and other life stressors altering amygdala-frontal circuitry [78] and suggest that maternal experiences of discrimination during pregnancy may be an additional stressor that can affect this circuit. The association of discrimination experiences with the connectivity of amygdala-frontal circuits remains when accounting for maternal perceived stress and depressive symptoms, which are also known to affect these circuits [71–77]. These findings suggest a specificity to experiences of discrimination that are independent of other stressors. Prior studies have clarified that discrimination and acculturation affect psychological well-being in adults, independent of stress or depression [25]. Our finding would be in line with that literature. While many intercorrelated stressors and experiences may alter amygdala-frontal connectivity, their causal interactions are not understood. Studies understanding and delineating their shared and unique effects are needed.

The associations between amygdala connectivity, discrimination, and acculturation may also have emotional processing implications in later development. The amygdala contributes to the processing of affective visual information [79]. Indeed, emotional valence moderates the “other-race effect”; positive valence reduces this effect [80]. The amygdala likely contributes to this interaction [81]. Likewise, frontal networks act top-down to down-regulate the amygdala’s neural responses [82]. Given the importance of emotion processing and regulation in later mental health disorders [83], studies investigating the associations between prenatal discrimination and acculturation, infant brain measures, and later emotional regulation (as well as potential mediating effects) should be explored.

We observed insignificant associations of discrimination and acculturation stressors with fetal and birth outcomes, diverging from previous results. Study design and maternal characteristics may explain differences. Observed effect sizes were small to medium, suggesting that significant results would be observable with a larger sample, in line with previous studies [11, 14, 15, 23, 24]. Similarly, our sample consisted only of minoritized women. Thus, we did not compare outcomes to a reference population (typically, non-Hispanic white women) as in other studies. Finally, our sample consisted exclusively of adolescents. Maternal age itself is a potent variable affecting fetal and birth outcomes. Younger maternal age and mothers from a minoritized background are well-known predictors of worse fetal and birth outcomes [84]. The risk of worse outcomes might not linearly scale with discrimination and acculturation experienced by these individuals but rather lies within the inherent characteristics of this population.

Although we hypothesized that other known stress-related factors would be distinct from acculturation and discrimination, we did not specify how they might relate. Prior work indicates that acculturation could play a role in moderating the experience of discrimination. For example, as individuals become more integrated into their host society, their perception of discrimination may intensify [85, 86]. The experience of acculturative stress has also been shown to lessen with the presence or lack of familial social support and socioeconomic status [87]. In addition to our findings of no significant differences in acculturation between those who experienced discrimination and those who did not, future research could explore social support and an expanded number of socioeconomic factors.

Clarifying the biological mechanisms by which maternal discrimination and acculturation affect the developing fetal brain is also a potential next step. Many stressors can have similar immediate physiological responses in the mother and fetus (i.e., increased heart rate) but different longer-term physiological responses. As another example, perceived stress and depression can have a differential impact on maternal sleep, inflammation, and eating/nutrition, which in turn have unique downstream effects on the fetus [45, 46]. Nevertheless, even though exposure to various forms of maternal distress (perceived stress, depression, anxiety), discrimination, and acculturation may have different outcomes for the developing fetus, they may have shared biological mediators [47]. The maternal hypothalamic-pituitary-adrenal (HPA) axis—the central stress response system [88]—is a critical pathway for maternal stressors to affect the developing fetus [89]. Future work should clarify how various stressors influence the biological pathways that alter infant connectivity.

Our study had several critical strengths. Our sample consisted entirely of women of color/underrepresented individuals, allowing for data collection from a population that may experience discrimination and acculturation and that is underrepresented in neuroimaging and biomedical studies. Also, our maternal sample consisted of adolescent pregnant women—another less studied population. Our unique approach to measuring acculturation combines two scales, the SASH, and AHIMSA. These measures have good construct validity [90, 91] and go beyond prior studies’ utilization of language as a primary determinant of acculturation. Furthermore, our study focuses on discrimination as separate from other stressors, an essential distinction given our sample and the current social climate.

Our study also had several limitations. Firstly, acculturation studies have limits concerning generalizability, given that every culture is different and people within each ethno-racial group are not homogenous. Our sample of convenience was predominantly Hispanic adolescent participants and is therefore only representative of this population. Future studies with more extensive and diverse samples will help better characterize the effects of prenatal experience of discrimination and acculturation. The acculturation measures, SASH and AHIMSA, used in the current study are tailored to acculturation in Hispanic individuals. As 12% of our sample were non-Hispanic, those individuals may not relate to all the items on the scales. Therefore, the scales might be less valid. Future studies should include measures that are more inclusive of other minoritized groups. We only had a single measure of SES rather than comprehensive measures of structural and systemic barriers (e.g., neighborhood-level characteristics). As in prior studies, these factors are essential and likely contributed to our findings [87]. Our infant sample was small and a majority male. As a result, it was underpowered to detect sex differences. Also, the literature on the neural correlates of discrimination, acculturation, and ethno-racial processing in adults involves more than just the amygdala [36–38]. Examining other brain regions is a needed future step. Finally, our study utilized a secondary data analysis. We obtained fetal and birth outcome data from electronic health records. Recordings of anthropometric variables can vary by medical personnel. Future studies should employ a prospective study design with measures of microaggressions, stigma, family support, and other structural factors to better understand the associations of discrimination and acculturation stress with infant outcomes.

It is also important to note that we do not have information regarding the length of time each participant has been in the United States (e.g., first versus other generation) or complementary qualitative interviews to ascertain the extent to which acculturation or discrimination is experienced as a stressor. Prior studies suggest acculturation experiences as stressors, and the impact on health is complex and can vary greatly [10]. Given that there was no significant association between acculturation and the distress measures (e.g., perceived stress); no significant difference in acculturation style between those who experienced discrimination and those who did not; and average report of lower levels of assimilation, separation, and marginalization on the AHIMSA, it is possible that our study sample experienced lower levels of acculturation-related stress Lastly, the self-identification of race of Latinx populations is complex. For example, many self-identify as indigenous [92]. A high proportion of individuals in our sample reported indigenous race, so we needed more variation to conduct analyses at the race level.

## Conclusion

As our society strives for greater cultural inclusivity and sensitivity, we must become aware of the effects that the experience of acculturation and discrimination have on pregnant women and their infants. Our findings suggest that maternal prenatal discrimination and acculturation could be additional stressors associated with neonatal functional connectivity of the amygdala. Even though our study is with infants, it is essential to consider our findings in the larger context of the adult literature related to discrimination and acculturation [10, 26–35] as these infants will continue to grow and the priming of the maternal experience will become integral to the cumulative exposure from their own personal experience of discrimination and acculturation throughout their life. Studies would benefit from including discrimination and acculturation to further understand the effects of these stressors on brain development of future generations. Longitudinal designs that consider the downstream or long-term effects, such as accelerated aging, are needed [27, 28].

### Supplementary information

Supplement
