# Correction: The effects of experience of discrimination and acculturation during pregnancy on the developing offspring brain

**DOI:** 10.1038/s41386-024-01798-2

**Published:** 2024-01-11

**Authors:** Marisa N. Spann, Kiarra Alleyne, Cristin M. Holland, Antonette Davids, Arline Pierre-Louis, Claire Bang, Victoria Oyeneye, Rebecca Kiflom, Eileen Shea, Bin Cheng, Bradley S. Peterson, Catherine Monk, Dustin Scheinost

**Affiliations:** 1 Vagelos College of Physicians and Surgeons, Columbia University, New York, NY USA; 2 New York State Psychiatric Institute, New York, NY USA; 3 Columbia University Mailman School of Public Health, New York, NY USA; 4 Rutgers University, Newark, NJ USA; 5 Columbia University, New York, NY USA; 6 Institute for the Developing Mind, Children’s Hospital Los Angeles, Los Angeles, CA USA; 7 Department of Psychiatry, Keck School of Medicine, University of Southern California, Los Angeles, CA USA; 8 Yale University School of Medicine, New Haven, CT USA

**Keywords:** Cognitive neuroscience, Risk factors

Correction to: *Neuropsychopharmacology* 10.1038/s41386-023-01765-3, published online 15 November 2023

The article “The effects of experience of discrimination and acculturation during pregnancy on the developing offspring brain”, written by Marisa N. Spann, Kiarra Alleyne, Cristin M. Holland, Antonette Davids, Arline Pierre-Louis, Claire Bang, Victoria Oyeneye, Rebecca Kiflom, Eileen Shea, Bin Cheng, Bradley S. Peterson, Catherine Monk and Dustin Scheinost, was originally published Online First without Open Access. After publication in volume 49, issue 2, page 476–485, the author decided to opt for Open Choice and to make the article an Open Access publication. Therefore, the copyright of the article has been changed to © The Author(s) 2024 and the article is forthwith distributed under the terms of the Creative Commons Attribution 4.0 International License, which permits use, sharing, adaptation, distribution and reproduction in any medium or format, as long as you give appropriate credit to the original author(s) and the source, provide a link to the Creative Commons licence, and indicate if changes were made.

The images or other third party material in this article are included in the article’s Creative Commons licence, unless indicated otherwise in a credit line to the material. If material is not included in the article’s Creative Commons licence and your intended use is not permitted by statutory regulation or exceeds the permitted use, you will need to obtain permission directly from the copyright holder.

To view a copy of this licence, visit http://creativecommons.org/licenses/by/4.0/.

