# Supplementary material for: The effects of experience of discrimination and acculturation during pregnancy on the developing offspring brain
Source: Neuropsychopharmacology. 2023 Nov 15;49(2):476–85. doi: 10.1038/s41386-023-01765-3 (PMC10724278; doi:10.1038/s41386-023-01765-3)
Supplement: Supplementary file 1 — Supplement [file 41386_2023_1765_MOESM1_ESM.docx]

**Supplemental Methods**

***Demographics*.** A previously validated psychosocial questionnaire was administered to each participant to obtain demographic information such as ethnicity, education, and family structure. Prenatal electronic health records were reviewed to determine birth outcomes including gestational age at birth, birth weight, infant length, Apgar score, and delivery data. Obstetrical and neonatal records were reviewed to extract information on the postmenstrual age (PMA), Apgar scores, gestational age at birth, birth weight, and delivery data from medical records. PMA is defined as the time elapsed between the first day of the pregnant woman’s last normal menstrual period and the time of the MRI scan of their infant. Apgar is an assessment performed on the neonate following birth at 1- and 5-minutes following birth. The score includes breathing effort, heart rate, muscle tone, reflexes, and skin color. The total score ranges from 1 to 10. Gestational age at birth was determined from the medical record for dates of ultrasound examinations and last reported menstrual cycle.

New York City is known for its large cultural enclaves, which can be segregated communities of individuals with similar cultural backgrounds. This segregation is well studied in the NYC public school system, which could influence adolescent participants, as well as, at the neighborhood level [1]. In this sense, an individual may experience two communities. The first being a local community that reflects the culture of one’s ethnic or racial background and potentially offers social support which research has shown to mediate potential negative effects of acculturation [2,3]. The second being a broader community reflecting the host country’s culture. How these communities interact to affect an individual’s experience of acculturation was not specifically measured in this study, which is a limitation. Acculturation measures can be administered to individuals without knowing the source of acculturation and regardless of whether one is a recent immigrant or they are second, third, or more generations in the respective country [4,5].

***Acculturation.*** Acculturation can include any individual changes that occur due to migration and subsequent interactions with differing cultures [6,7]. Level of acculturation was obtained with two measures—the Short Acculturation Scale for Hispanics (SASH) and the Acculturation, Habits, and Interests Multicultural Scale for Adolescents (AHIMSA). Acculturation in a population can be represented in a unidimensional model of high versus low assimilation and Berry's multidimensional model. Berry's multidimensional model outlines four categories: acquiring or rejecting the host culture and retaining or rejecting the home culture[8]. These categories are integration (the identification with both cultures), assimilation (identification with the host culture), separation (identification with home culture), and marginalization (the identification with neither home nor host culture). The AHIMSA is based on Berry's model, while the SASH is a unidimensional model.

The SASH measures behavioral factors previously associated with acculturation, including language use, media preference, and ethnic social relations [9]. This measure has 3 subscales and 12-items. The language use subscale asks participants which language they use when reading, speaking, with a child, at home, thinking, and with friends. The media use subscale asks participants to identify the language with which they watch and listen to TV programs, radio programs and movies, and the ethnic social relations subscale asks participants who they spend time with and relate to most. Total scores range from 12-60 with higher scores indicating greater levels of acculturation. SASH has been psychometrically examined and has been found to be both a useful and valid measure of acculturation [10].

The AHIMSA is a measure of acculturation [11] for adolescents. It has 8 questions that address adolescents’ social experiences, such as who they are friends with and where their TV shows come from. There are four response categories which each indicate a varying level of acculturation: a. The United States (Assimilation), b. The country my family is from (Separation), c. Both (Integration), d. Neither (Marginalization). Four scores are given based on the four response categories or orientations, and the score for each orientation ranges from 0-8.

***Discrimination***. The Experience of Discrimination (EOD) instrument measured self-reported experiences of ethnoracial discrimination and was scored by counting the number of situations for which a participant experienced discrimination [12]. The validity and reliability of the measure has been tested among a diverse population in order to confirm its psychometric properties [13]. The EOD measures lifetime frequency of discrimination.

***Perceived Stress.*** The Perceived Stress Scale (PSS) is a commonly used measure for subjective stress with good internal and test-retest reliability [14]. It measures perceived stress by having participants rate their feelings related to potentially stressful events that had occurred within the last month. There are 14 items in which 7 of the items are negative and 7 of the items are positive. Each item was rated on a five-point Likert-type scale in which 0 indicated never and 4 indicated very often. The 7 positive items were then reverse coded so that all the scores could be summed. Higher scores demonstrated greater stress. The PSS covers perceived stress over the last month.

***Depression.*** The Reynolds Adolescent Depression Scale (RADS) is a self-report measure that reflects symptoms of depressive disorders in adolescents [15]. The 30–item scale has four subscales: Dysphoric Mood, Anhedonia/Negative Affect, Negative Self-Evaluation, and Somatic Complaints. Item scores from each subscale are summed to create a composite depression score that ranges from 30-120, higher scores are an indication of more severe depressive symptoms. RADS is a commonly used measure due to its proven validity and reliability [16]. The RADS cover symptoms over the past week.

***Childhood trauma.*** The Childhood Trauma Questionnaire (CTQ) is a 28-item self-report inventory that measures the severity of different types of childhood trauma [17]. Reponses are measures on a 5-point Likert-type scale (1=never true, 5=very often true). It has five subscales that measure childhood trauma: emotional abuse, physical abuse, sexual abuse, emotional neglect, and physical neglect. Scores fall into four categories: none to low trauma exposure, low to moderate trauma exposure, moderate to severe trauma exposure, and severe to extreme trauma exposure for each scale. The CTQ covers lifetime trauma prior to age 18 years.

***Imaging procedures*.** Infants were fed, swaddled, and acclimated to the scanning environment and scanner noise by listening to a tape recording of the scanner sounds played before each pulse sequence. The infants were given time to fall asleep without using sedatives while lying on the scanner bed before the start of each sequence. To dampen scanner noise, infants wore foam and wax ear plugs and ear shields (Natus Medical Inc., San Carlos, CA). MRI-compatible EKG leads were placed on the infant's chest, and pulse oximetry sensor was placed on the infant's toe. Heart rate and oxygen saturation were continuously monitored during the scan (InVivo Research, Orlando, FL).

***Imaging parameters:*** Images were obtained using a 3 Tesla General Electric (GE) Signa MRI scanner (Milwaukee, Wisconsin) and an 8-channel head coil. High resolution anatomical T2-weighted images were acquired using a 2D, multiple-shot, fast spin echo pulse sequence that employed PROPELLER (Periodically Rotated Overlapping Parallel Lines with Enhanced Reconstruction) to reduce motion artifacts in reconstructed MR images [18]: repetition time (TR)=10,000 ms; echo time (TE)=130 ms; echo train length (ETL)=32; matrix size=192×192; field of view (FOV)=190×190 mm; phase FOV=100%; slice thickness=1.0mm; number of excitations (NEX)=2. The spatial resolution of the T2-weighted images was 1mm^3^. Functional images were acquired using a standard echo-planar imaging sequence: TR=2,200 ms; TE=30 ms; matrix size=64×64; FOV=190×190 mm; phase FOV=100%; slice thickness=5.0mm, contiguous; number of slices=24; bandwidth=7812.5 Hz. Although the number of runs acquired varied per participant due to compliance, the median of six runs of 102 volumes (3 minutes 44.4 sec each) were obtained for each infant.

***Common space registration.*** First, anatomical images were skull stripped using FSL (https://fsl.fmrib.ox.ac.uk/fsl/) and any remaining non-brain tissue was manually removed. All further analyses was performed using BioImage Suite [19] unless otherwise specified. Anatomical images were linearly aligned to a single infant anatomical scan from an independent study [20] using a 12 parameter affine registration by maximizing the normalized mutual information between images. Next, anatomical images were non-linearly registered to an evolving group average template in an iterative fashion using a previously validated algorithm [21]. This algorithm iterates between estimating a local transformation to align individual brains to a group average template and creating a new group average template based on the previous transformations. The local transformation was modeled using a free-form deformation (FFD) parameterized by cubic B-splines. This transformation deforms an object by manipulating an underlying mesh of control points. The deformation for voxels in between control points was interpolated using B-splines to form a continuous deformation field. Positions of control points were optimized using a conjugate gradient descent to maximize the normalized mutual information between the template and individual brains. After each iteration, the quality of the local transformation was improved by increasing the number of control points and decreasing the spacing between control points to capture a more precise alignment. A total of 5 iterations were performed with decreasing control point spacings of 15 mm, 10 mm, 5 mm, 2.5, and 1.25 mm. To help prevent local minimums during optimization, a multi-resolution approach was used with three resolution levels at each iteration. Finally, functional images were rigidly aligned to the anatomical images.

All transformation pairs were calculated independently and combined into a single transform, warping the single participant results into common space. This single transformation allows the individual participant images to be transformed to the common space with only one transformation, thereby reducing interpolation error.

***Connectivity processing.*** Fifty infants were scanned within the first weeks of postmenstrual life (mean=42.3; SD=1.6 weeks postmenstrual age). 38 infants had usable fMRI data and complete prenatal data. Motion correction was performed using SPM8 (http://www.fil.ion.ucl.ac.uk/spm/). Images were warped into 3 mm^3^ common space using the non-linear transformation and cubic interpolation (see Supplement). Next, images were iteratively smoothed until the smoothness of an image had a full-width half maximum of approximately 8 mm [48] using AFNI’s 3dBlurToFWHM (<http://afni.nimh.nih.gov/afni/>). Iteratively smoothing to a set smoothness reduces the smoothing applied to the image (which helps resolve smaller structures like the amygdala) and motion-related confounds. Several covariates of no interest were regressed from the data, including linear and quadratic drifts, mean cerebral-spinal-fluid (CSF) signal, mean white-matter signal, and mean gray matter signal. For additional control of possible motion-related confounds, a 24-parameter motion model (including six rigid-body motion parameters, six temporal derivatives, and these terms squared) was regressed from the data. The functional data were temporally smoothed with a Gaussian filter (approximate cutoff frequency=0.12Hz).

***Motion Analysis.*** As motion and amount of data for analysis effects functional connectivity measures [22,23], we employed a strict inclusion criterion that participants had at least 2 runs of data with an average frame-to-frame motion of less than 0.1 mm. For infants with more than 2 runs with good movement, we selected the 2 runs with the least frame-to-frame motion. We detected no significant correlations between motion and maternal experience of discrimination and acculturation (r’s<0.15, p’s>0.4). Further, as described above, we employed global signal regression, a 24-parameter motion model regression, and uniform smoothing to minimize motion confounds not accounted for by our inclusion criteria.


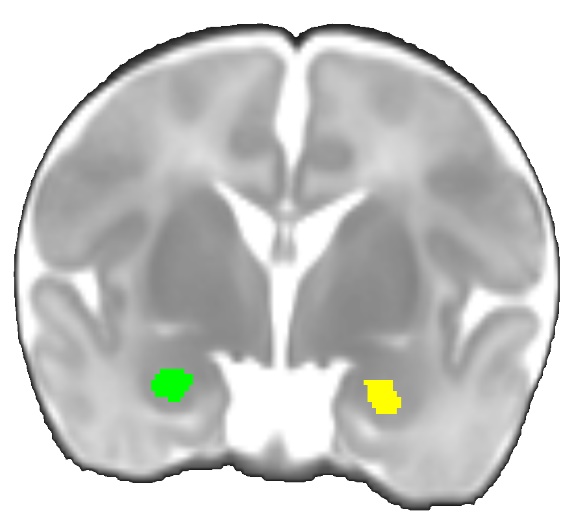


**Figure S1: Left (shown in green) and right (shown in yellow) amygdala seeds.** For the main analyses, these seeds were combined into a single seed for whole-brain connectivity.

**Supplemental Results**

For face validity, we investigated if language use—a standard proxy for acculturation—correlates with the acculturation factors. The ASSIMILATION-INTEGRATION factor was higher in women who did not declare Spanish as either a primary or secondary language compared to women who declared Spanish as their primary language (t= 5.08, p<0.001, df=80, Cohen’s d=1.13) or secondary language (t=-4.7, p<0.001, df=112, Cohen’s d=0.89). In addition, women who declared Spanish as their primary language had lower scores on the ASSIMILATION-SEPARATION factor compared to women who either declared Spanish as their second language (t=-8.76, p<0.001, df=132, Cohen’s d=1.52) and women who did not declare Spanish as a primary or secondary language (t=-6.08, p<0.001, df=80, Cohen’s d=1.36).

There is no difference in the frequency of a zero EOD score between the Hispanic group and the non-Hispanic group (Chi-square=1.45, p=0.23). In the EOD > 0 group, EOD correlated with RADS (r=0.32; p=0.004), PSS (r=0.27; p=0.02), and CTQ (r=0.29; p=0.01). For the AHIMSA, in the Assimilation >0 group, Assimilation correlated with Separation (r=-0.35; p<0.001), Integration (r=-0.81; p<0.001), and SASH (r=0.29; p=0.002). In the Separation >0 group, Separation correlated with Assimilation (r=-0.35; p<0.001), Integration (r=-0.57; p<0.001), and SASH (r=0.50; p=0.002). In the Integration >0 group, Integration correlated with Assimilation (r=-0.81; p<0.001), Separation (r=-0.57; p<0.001), and SASH (r=-0.50; p<0.001). In the Integration >0 group, Integration correlated with Assimilation (r=-0.81; p<0.001), Separation (r=-0.57; p<0.001), and SASH (r=-0.50; p<0.001). In the Marinization >0 group, Marinization correlated with Integration (r=-0.48; p<0.03). In sum, it appears that for those experience discrimination there are linear associations with other stressors like depression, perceived stress, and childhood trauma. In contrast, for the AHIMSA, only correlations between the items and the other acculturation measure (SASH) were observed.


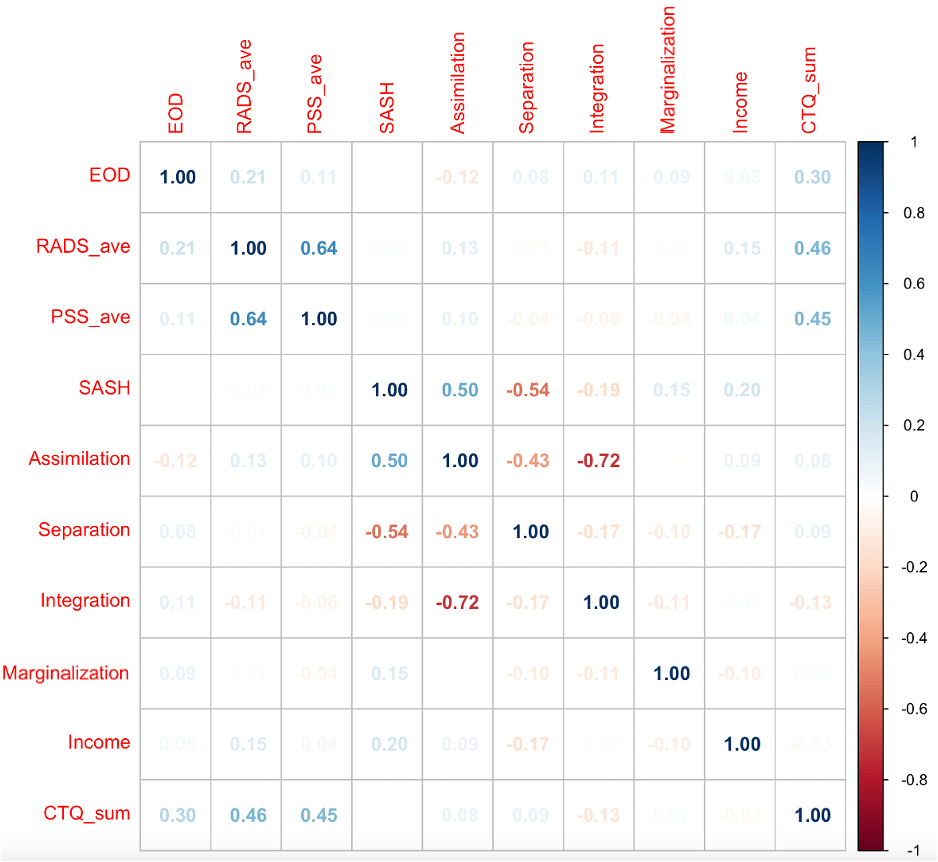


**Figure S2: Correlation matrix of study variables. |r’s|>0.25 are significant after Bonferroni correction.**

Abbreviations: SASH, Short Acculturation Scale for Hispanics; EOD, Experience of Discrimination; PSS, Perceived Stress Scale; CTQ, Childhood Trauma Questionnaire; RADS, Reynold’s Adolescent Depression Scale.


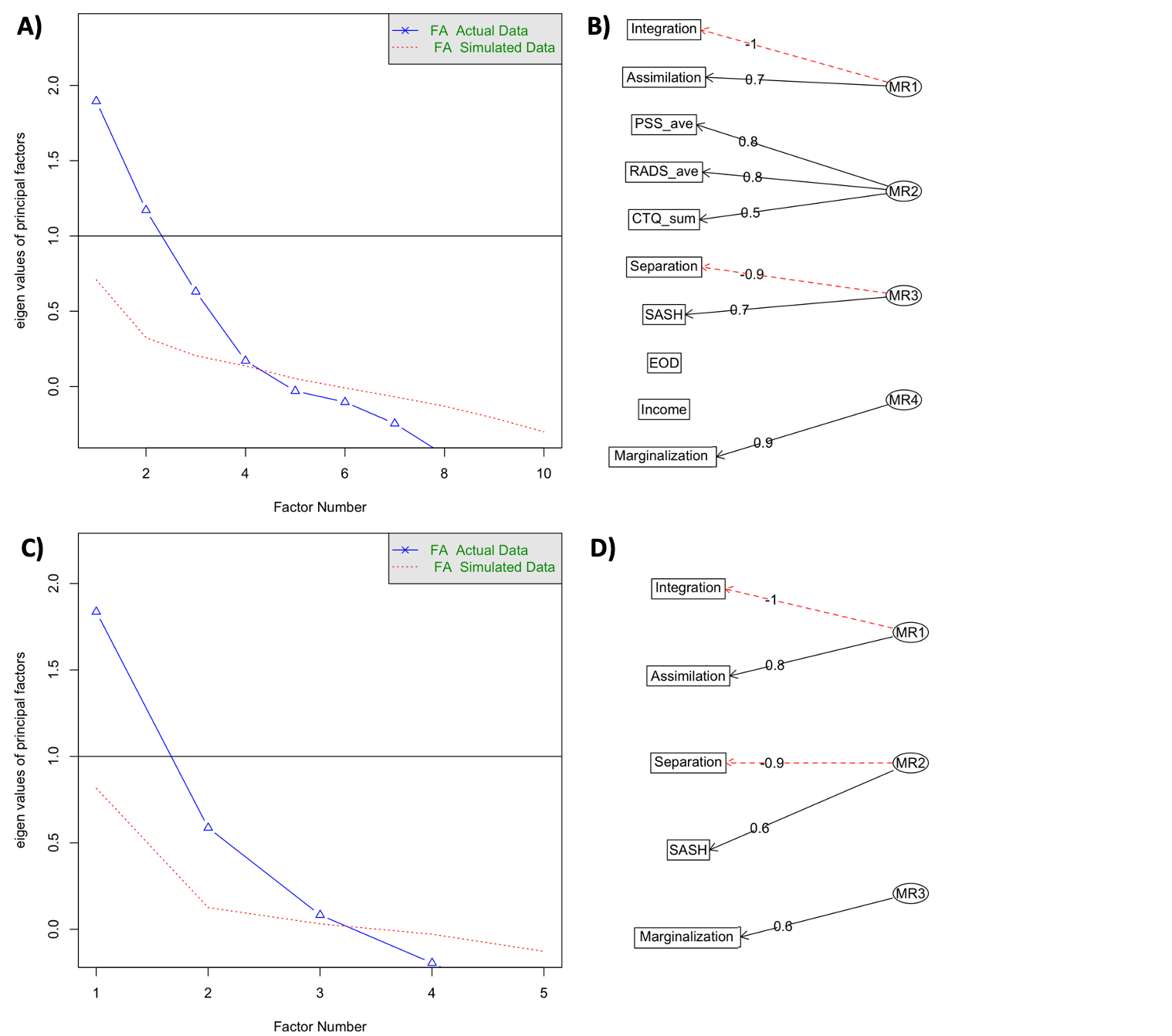


**Figure S3: Factor analysis results. A)** For the factor analysis of experienced distress, acculturation, and discrimination during pregnancy, four factors were determined to be the optimal solution. **B)** Stress clustered into a single factor, while acculturation clustered into three factors. Discrimination did not cluster into a factor. **C)** For the factor analysis of experienced acculturation during pregnancy, three factors were determined to be the optimal solution. **D)** Similar acculturation factors were found with this reduced model compared to the full model that included distress and discrimination. Solid black lines in **B)** and **C)** indicate a positive loading of a measure on a factor, while red dot lines indicate a negative loading. Abbreviations: SASH, Short Acculturation Scale for Hispanics; EOD, Experience of Discrimination; PSS, Perceived Stress Scale; CTQ, Childhood Trauma Questionnaire; RADS, Reynold’s Adolescent Depression Scale.

| **Table S1: Item and loadings for the first factor analysis** | | | | |
| --- | --- | --- | --- | --- |
| Item | Factor 1 | Factor 2 | Factor 3 | Factor 4 |
| EOD | 0.13 | 0.34 | 0.06 | 0.12 |
| RADS | 0.42 | 0.69 | 0.15 | 0.02 |
| PSS | 0.42 | 0.63 | 0.17 | 0.05 |
| SASH | 0.53 | -0.29 | 0.27 | 0.12 |
| Assimilation | 0.90 | -0.36 | -0.10 | -0.22 |
| Separation | -0.38 | 0.41 | -0.74 | -0.20 |
| Integration | -0.71 | 0.12 | 0.70 | 0.08 |
| Marginalization | 0.09 | -0.08 | -0.29 | 0.89 |
| Income | 0.19 | -0.03 | 0.18 | -0.10 |
| CTQ | 0.27 | 0.52 | 0.03 | 0.02 |
| Abbreviations: SASH, Short Acculturation Scale for Hispanics; EOD, Experience of Discrimination; RADS, Reynold’s Adolescent Depression Scale; PSS, Perceived Stress Scale; CTQ, Childhood Trauma Questionnaire; | | | | |

| **Table S2: Item and loadings for the second factor analysis** | | | |
| --- | --- | --- | --- |
| Item | ASSIMILATION-INTEGRATION | ASSIMILATION-SEPARATION | MARGINALIZATION |
| SASH | 0.57 | -0.30 | 0.08 |
| Assimilation | 0.97 | 0.06 | -0.24 |
| Separation | -0.48 | 0.86 | -0.16 |
| Integration | -0.74 | -0.67 | 0.06 |
| Marginalization | 0.16 | 0.22 | 0.95 |
| Abbreviations: SASH, Short Acculturation Scale for Hispanics | | | |

| **Table S3: Volume of significant clusters** | |
| --- | --- |
| ***Brain Region*** | ***Cluster Size*** |
| ASSIMILATION-SEPARATION clusters | |
| Left Fusiform | 610 mm^3^ |
| Right Fusiform | 461 mm^3^ |
| Experience of Discrimination clusters | |
| Medial Prefrontal Cortex | 810 mm^3^ |
| Medial Prefrontal Cortex | 1026 mm^3^ |
| Left Fusiform | 592 mm^3^ |

| **Table S4. Associations between maternal experience of discrimination and acculturation and fetal growth outcomes** | | | | |
| --- | --- | --- | --- | --- |
| **Discrimination and acculturation factors** | **Fetal Growth Outcomes** | | | |
|  | **Head Circumference** | | **Bi-Parietal**  **Diameter** | |
|  | ***T*** | **p-value** | ***t*** | **p-value** |
| ASSIMILATION-INTEGRATION | 0.50 | 0.62 | -2.06 | 0.04 |
| ASSIMILATION-SEPARATION | 0.67 | 0.50 | 1.30 | 0.20 |
| MARGINALIZATION | -0.99 | 0.33 | -0.84 | 0.40 |
| Experience of Discrimination | 0.75 | 0.46 | 1.15 | 0.25 |

| **Table S5. Associations between maternal experience of discrimination and acculturation and birth outcomes** | | | | |
| --- | --- | --- | --- | --- |
| **Discrimination and acculturation factors** | **Birth Outcomes** | | | |
|  | **Gestational Age at Birth** | | **Apgar Score at**  **5 minutes** | |
|  | ***T*** | **p-value** | ***t*** | **p-value** |
| ASSIMILATION-INTEGRATION | -0.04 | 0.97 | -1.98 | 0.05 |
| ASSIMILATION-SEPARATION | -2.14 | 0.03 | -1.48 | 0.12 |
| MARGINALIZATION | 0.15 | 0.88 | -0.70 | 0.49 |
| Experience of Discrimination | -1.53 | 0.13 | -0.77 | 0.44 |

**References**

1 Kucsera J, Orfield G. New York State’s extreme school segregation: Inequality, inaction and a damaged future. 2014.

2 Espeleta HC, Beasley L, Bohora S, Ridings LE, Silovsky JF. Depression in Latina mothers: Examining the roles of acculturation, enculturation, social support, and family resources. Cultural diversity and ethnic minority psychology. 2019;25(4):527.

3 Rivera FI. Contextualizing the experience of young Latino adults: Acculturation, social support and depression. Journal of Immigrant and Minority Health. 2007;9:237-44.

4 Amer MM, Hovey JD. Examination of the impact of acculturation, stress, and religiosity on mental health variables for second-generation Arab Americans. Ethn Dis. 2005;15(1 Suppl 1):S1-111-2.

5 Hovey JD, King CA. Acculturative stress, depression, and suicidal ideation among immigrant and second-generation Latino adolescents. J Am Acad Child Adolesc Psychiatry. 1996;35(9):1183-92.

6 Gibson MA. Immigrant adaptation and patterns of acculturation. Human development. 2001;44(1):19-23.

7 Garcia AF, Wilborn, K., & Mangold, D. L. . The Cortisol Awakening Response Mediates the Relationship Between Acculturative Stress and Self-Reported Health in Mexican Americans. Annals of behavioral medicine: a publication of the Society of Behavioral Medicine. 2017;51(6):787–98.

8 Berry JW, Padilla AM. Acculturation: Theory, models and some new findings. Acculturation as varieties of adaption. 1980;9:25.

9 Marin G, Sabogal F, Marin BV, Otero-Sabogal R, Perez-Stable EJ. Development of a Short Acculturation Scale for Hispanics. Hispanic Journal of Behavioral Sciences. 1987;9(2):183-205.

10 Ellison J, Jandorf L, Duhamel K. Assessment of the Short Acculturation Scale for Hispanics (SASH) among low-income, immigrant Hispanics. J Cancer Educ. 2011;26(3):478-83.

11 Unger JG, P.; Shakib, S.; Ritt-Olson, A.; Palmer, P.; and Johnson, C. The AHIMSA Acculturation Scale: A New Measure of Acculturation for Adolescents in a Multicultural Society. Journal of Early Adolescence. 2002;22(3):225-51.

12 Krieger N. Racial and gender discrimination: risk factors for high blood pressure? Social science & medicine. 1990;30(12):1273-81.

13 Krieger N, Smith K, Naishadham D, Hartman C, Barbeau EM. Experiences of discrimination: validity and reliability of a self-report measure for population health research on racism and health. Social science & medicine. 2005;61(7):1576-96.

14 Cohen S, Kamarck T, Mermelstein R. A global measure of perceived stress. J Health Soc Behav. 1983;24(4):385-96.

15 Reynolds WM. (Western Publishing Services, Torrance, CA, 1987).

16 Osman A, Gutierrez PM, Bagge CL, Fang Q, Emmerich A. Reynolds adolescent depression scale-second edition: a reliable and useful instrument. J Clin Psychol. 2010;66(12):1324-45.

17 Bernstein DP, Ahluvalia T, Pogge D, Handelsman L. Validity of the Childhood Trauma Questionnaire in an adolescent psychiatric population. J Am Acad Child Adolesc Psychiatry. 1997;36(3):340-8.

18 Pipe JG. Motion correction with PROPELLER MRI: application to head motion and free-breathing cardiac imaging. Magn Reson Med. 1999;42(5):963-9.

19 Joshi A, Scheinost D, Okuda H, Belhachemi D, Murphy I, Staib LH, et al. Unified framework for development, deployment and robust testing of neuroimaging algorithms. Neuroinformatics. 2011;9(1):69-84.

20 Scheinost D, Kwon SH, Shen X, Lacadie C, Schneider KC, Dai F, et al. Preterm birth alters neonatal, functional rich club organization. Brain Struct Funct. 2016;221(6):3211-22.

21 Scheinost D, Kwon SH, Lacadie C, Vohr BR, Schneider KC, Papademetris X, et al. Alterations in Anatomical Covariance in the Prematurely Born. Cerebral Cortex. 2017;27(1):534-43.

22 Noble S, Spann MN, Tokoglu F, Shen X, Constable RT, Scheinost D. Influences on the Test-Retest Reliability of Functional Connectivity MRI and its Relationship with Behavioral Utility. Cereb Cortex. 2017;27(11):5415-29.

23 Van Dijk KR, Sabuncu MR, Buckner RL. The influence of head motion on intrinsic functional connectivity MRI. Neuroimage. 2012;59(1):431-8.
